# Complete Genome and Plasmid Sequences of Escherichia coli Type Strain ATCC 11775

**DOI:** 10.1128/MRA.00046-19

**Published:** 2019-02-28

**Authors:** Taylor D. Wadley, Piroon Jenjaroenpun, Thidathip Wongsurawat, David W. Ussery, Intawat Nookaew

**Affiliations:** a Department of Biomedical Informatics, College of Medicine, University of Arkansas for Medical Sciences, Little Rock, Arkansas, USA; b Department of Physiology and Biophysics, College of Medicine, University of Arkansas for Medical Sciences, Little Rock, Arkansas, USA; University of Rochester School of Medicine and Dentistry

## Abstract

Escherichia coli ATCC 11775 is a strain that was identified in 1941 and is now considered a type strain for the species. We present here the complete genome sequence for E. coli ATCC 11775. The genome was sequenced using Oxford Nanopore Technologies products and had 4,903,501 and 131,333 nucleotides of sequence length of the individual chromosome and plasmid, respectively.

## Abstract (precis)

Escherichia coli ATCC 11775 is a strain that was identified in 1941 and is now considered a type strain for the species. We present here the complete genome sequence for E. coli ATCC 11775.

## ANNOUNCEMENT

Escherichia coli is a Gram-negative rod-shaped bacterium found in the large intestine of endotherms. It is one of the most heavily studied organisms ever. We sequenced E. coli type strain ATCC 11775 (= DSM 30083 = U5/41), which was first isolated from a Danish patient’s urine in 1941. The strain has been used as a reference in the years since (1). The current submission for ATCC 11775 in NCBI (submission number GCF_000690815.1) is broken into three contigs with one plasmid (1). Therefore, we aim to achieve a complete genome of the E. coli type strain that can be used in the community. We used Nanopore MinION sequences along with the Illumina data to fully assemble the DSM 30083 genome and one plasmid.

**FIG 1:**
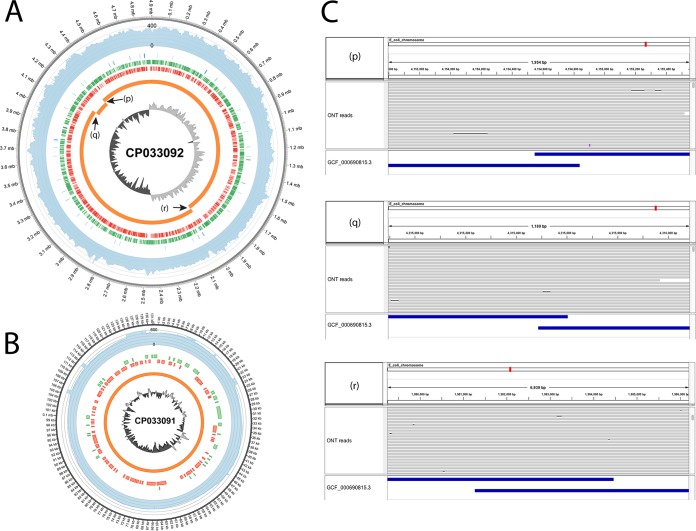
Chromosome (A) and plasmid (B) comparison of assembled Escherichia coli ATCC 11775 results between this study and that of Meier-Kolthoff et al. (1). From the outside in, (i) the assembled genome from this study (with the genome coordinate), (ii) ONT read coverage depth plot (light blue), (iii) rRNA and tRNA locations (blue), (iv) positive-strand open reading frame (ORF) locations (green), (v) negative-strand ORF locations (red), (vi) assembled genome from Meier-Kolthoff et al. (1) (orange), and (vii) GC-skew plot of the assembled genome from this study (light gray represents G-rich [positive value], dark gray represents non-G-rich [negative value]). The three overlap points of the contigs are shown in the p, q, and r locations. (C) Integrative Genomics Viewer (IGV) snapshots show that ONT reads span across the contigs of the GCF_000690815.1 genome on the repeat locations of p, q, and r as shown in panel A. Top, chromosome location; middle, ONT read alignment; bottom, GCF_000690815.1 contigs.

We purchased the E. coli type strain from the American Type Culture Collection (ATCC). Cultures were grown in 25 ml lysogeny broth (LB) for 24 h. E. coli DNA was isolated from cultured cells using a Bactozol kit (Molecular Research Center) and cleaned up with Genomic DNA Clean & Concentrator (Zymo Research) according to the manufacturer’s protocol. The DNA was then used in DNA library preparation using the rapid barcoding kit (catalog number SQK-RBK004) from Oxford Nanopore Technologies (ONT). The principle of the kit is a transposase which simultaneously cuts template molecules and attaches barcoded tags to the cut ends. The target size of the input DNA is 20 to 40 kb. The DNA was then sequenced on a FLO-MN106 flow cell on a Mk1b MinION device in a MinKNOW version 1.11.5 software environment with the default settings. The data acquisition and preprocessing were performed with the criteria adopted from Jenjaroenpun et al. (2). First, FAST5 files were used to call corresponding bases by Albacore version 2.2.7 using the standard default configuration for the SQK-RBK004 library kit. After base calling was carried out, we obtained 317,997 reads in total corresponding to 2 Gb with an *N*_50_ value of 12,729 bp. The reads were filtered further by mean quality scores of greater than 9 and read lengths longer than 5,000 nucleotides. This resulted in 118,118 reads corresponding to 1.5 Gbases with an *N*_50_ value of 15,397 bp for *de novo* assembly. We obtained Illumina reads from Meier-Kolthoff et al. (1) (SRA accession number SRR3927310). The Illumina reads were subjected to adapter trimming and quality filtering by Trimmomatic (3) with the following parameters: illuminaclip, TruSeq3-PE.fa:2:30:10; leading, 3; trailing, 3; slidingwindow, 4:15; minlen, 36. More than 95% of the reads were retained for *de novo* assembly. The hybrid assembly of the read set (both ONT and Illumina reads) was performed using Unicycler version 0.4.4 (4) with default parameters. We obtained two circular contigs represented by a complete chromosome with a size of 4,903,501 bases and a plasmid with a size of 131,333 bases. The comparison between the assembled genome of this study and GCF_000690815.1 is shown in Fig. 1 as determined using Circos software (5). The four contigs of GCF_000690815.1 were nicely aligned on our assembled genome with an average nucleotide identity of 99.98%. The terminal of the three contigs of GCF_000690815.1 are overlaped as supported by ONT reads illustrated in Fig. 1C. Using DNAdiff of the MUMmer package (6), we found that 100% of the GCA_000690815.1 genome (without gaps) could be aligned on our assembled genome. The three contigs of the GCF_000690815.1 chromosome can be closed since the ONT reads span across the contig gaps.

### Data availability.

The complete chromosome and plasmid sequences of E. coli ATCC 11775 have been submitted to GenBank and are publicly available under the accession numbers CP033091 (plasmid) and CP033092 (chromosome). The raw data (FASTQ and fast5) of this work are available at the SRA database under accession number SRR8413645.
